# Only an Infirmary

**Published:** 1905-11-11

**Authors:** 


### "ONLY AN INFIRMARY."

The medical superintendent of the Hammersmith Infirmary reported to the Guardians of that parish that there were in that institution defects which were "dangerous to the inmates, inconvenient to the staff, and likely to lead to evil." And therefore he recommended certain alterations in the place. But one of the Guardians, Mr. Neighbour (evidently no relation to the Good Samaritan), opposed any improvement being made, on the ground that the institution in question was "only an infirmary," and, strange to say, his fellow-Guardians agreed that no improvements should be made. What Mr. Neighbour meant by "only an infirmary" we cannot guess; but if he meant that efficiency in either structure or management was less necessary in a poor-law infirmary than in a general hospital, we can only assume that he knows little of either. It is true that a considerable portion of an infirmary is devoted to chronic and incurable cases which an ordinary hospital would not receive, but to keep these patients in the comfort which their condition demands is as desirable in an infirmary as in any other hospital. And, more and more, infirmaries are being used for the reception of acute diseases. Our hospital accommodation, large as it is, is insufficient for the demands made upon it, and every metropolitan infirmary receives its share of accidents and injuries, and of all ordinary, and some uncommon non-infectious diseases. Indeed, the boundary line between hospital and poor-law infirmary is, so far as the cases treated go, but ill-defined. Therefore one cannot excuse the existence of defects "dangerous to the inmates and inconvenient to the staff" in an infirmary any more than in a hospital. But the difference is this: that whereas in a voluntary hospital such a report by the medical superintendent would at once lead to an effort at improving the conditions, even though doing so involved a fresh appeal to the public for funds, in the case of the poor-law infirmary, the matter is laid before the Guardians, and if they, having the fear of raising the rates before their eyes, decide to let things be, the institution may remain in an unsatisfactory condition for an indefinite time—probably until some unfortunate scandal comes to awaken public opinion. Voluntary contributors to a charity are much more alert than ratepayers to see that efficiency is maintained. Perhaps the *laissez-faire* policy of the Hammersmith Guardians will awaken the adherents of rate-supported hospitals to this fact.

